# The miR156/SPL module regulates apple salt stress tolerance by activating MdWRKY100 expression

**DOI:** 10.1111/pbi.13464

**Published:** 2020-09-04

**Authors:** Yue Ma, Hao Xue, Feng Zhang, Qiu Jiang, Shuang Yang, Pengtao Yue, Feng Wang, Yuanyan Zhang, Linguang Li, Ping He, Zhihong Zhang

**Affiliations:** ^1^ College of Horticulture Shenyang Agricultural University Shenyang China; ^2^ College of Horticulture Anhui Agricultural University Hefei China; ^3^ College of Bioscience and Biotechnology Shenyang Agricultural University Shenyang China; ^4^ College of Plant Protection Shenyang Agricultural University Shenyang China; ^5^ Shandong Institute of Pomology Taian Shandong China

**Keywords:** microRNA156, SQUAMOSA PROMOTER BINDING PROTEIN‐LIKE 13, WRKY100, apple, salt tolerance

## Abstract

Salt stress dramatically impedes plant growth and development as well as crop yield. The apple production regions are reduced every year, because of the secondary salt damage by improper fertilization and irrigation. To expand the cultivation area of apple (*Malus domestica*) and select salt‐resistant varieties, the mechanism of salt tolerance in apple is necessary to be clarified. The miR156/SPL regulatory module plays key roles in embryogenesis, morphogenesis, life cycle stage transformation, flower formation and other processes. However, its roles in the mechanisms of salt tolerance are unknown. In order to elucidate the mechanism of 156/SPL regulating salt stress in apple, we performed RLM‐5’ RACE and stable genetic transformation technology to verify that both mdm‐*MIR156a* and *MdSPL13* responded to salt stress in apple and that the latter was the target of the former. *MIR156a* overexpression weakened salt resistance in apple whereas *MdSPL13* overexpression strengthened it. A total of 6094 differentially expressed genes relative to nontransgenic apple plants were found by RNA‐Seq analysis of MdSPL13OE. Further verification indicated that MdSPL13 targeted the *MdWRKY100* gene promoter. Moreover, *MdWRKY100* overexpression enhanced salt tolerance in apple. Our results revealed that the miR156/SPL module regulates salt tolerance by up‐regulating *MdWRKY100* in apple. This study is the first to elucidate the mechanism underlying the miRNA network response to salt stress in apple and provides theoretical and empirical bases and genetic resources for the molecular breeding of salt tolerance in apple.

## Introduction

Salt stress can substantially inhibit plant growth and development (Nutan *et al*., 2020; Yang and Guo, 2018). Approximately 25% of the global cultivated land area is salinized and the problem continues to worsen because of climate change and desertification (Tuteja, 2007; Zhu, 2016). The perennial woody plant apple (*Malus domestica*) is a nonhalophyte (Flower *et al*., 2010). In apple production regions, secondary salt damage has been exacerbated by improper fertilization and irrigation and has adversely affected vegetative growth, reproductive development, and fruit yield and quality. Consequently, considerable economic losses have been incurred. Hence, elucidation of the molecular mechanisms of salt resistance in apple will facilitate the breeding and cultivation of salt‐tolerant varieties.

To adapt to their environment, plants have evolved various stress resistance mechanisms such as ABA synthesis, reactive oxygen species (ROS) clearance, ionic balance, ethylene response and mitogen‐activated protein kinase (MAPK) pathways that regulate downstream defence responses (Chen *et al*., 2012; Ding *et al*., 2015; Meng *et al*., 2018; Wei *et al*., 2008). Previous studies analysed the functions of individual stress resistance‐associated genes. However, the complex overlapping network of regulatory mechanisms underlying stress tolerance is poorly understood (Munns and Tester, 2008). Recently, high‐throughput sequencing and bioinformatics have disclosed transcription factors that participate in the salt stress response by regulating downstream gene expression (Chen *et al*., 2019; Nakashima *et al*., 2012; Wang *et al*., 2019a).

MicroRNAs (miRNAs) may also be involved in plant resistance to salt stress (Kumar *et al*., 2018; Navarrete *et al*., 2009). These are small noncoding RNAs that exert regulatory functions by binding complementary mRNA sequences, promote degradation or inhibit translation and silence corresponding genes (Catalanotto *et al*., 2016). The first miRNA identified in plants was miR156. It targets and regulates the SQUAMOSA PROMOTER BINDING PROTEIN‐LIKE (SPL) transcription factors (Cardon *et al*., 1997). The miR156/SPL regulatory module plays important roles in embryogenesis, developmental stage transformation, flowering time, flower formation and morphogenesis (Wang and Wang, 2015). It also regulates plant stress response. MiR156 overexpression in *Arabidopsis thaliana* and *Medicago sativa* (alfalfa) induced and sustained the expression of heat stress response genes. Thus, miR156/SPL regulation is associated with heat stress (Matthews *et al*., 2019; Stief *et al*., 2014). However, there are few reports on the roles of miR156/SPL in plant salt tolerance. MiR156 was up‐regulated under salt stress in *Arabidopsis* and *Saccharum officinarum* (sugarcane) but down‐regulated under salt stress in maize (Ding *et al*., 2009; Gentile *et al*., 2015; Liu *et al*., 2008). However, the mechanism by which miR156 regulates the plant salt stress response is unclear.

WRKY transcription factors participate in numerous biological pathways related to salt stress tolerance (Jiang *et al*., 2017). *AtWRKY46* regulated the ABA signalling pathway in *Arabidopsis* by controlling lateral root development under salt stress (Ding *et al*., 2015). *DgWRKY3* (*Dendranthema frandiflorum*) improved salt tolerance in tobacco by reducing H_2_O_2_ and malondialdehyde (MDA) accumulation (Liu *et al*., 2013). *GhWRKY41* (*Gossypium hirsutum*) enhanced salt and drought tolerance in transgenic tobacco by regulating stomatal conductance and ROS levels (Chu *et al*., 2015). *GhWRKY34* lowered the Na^+^/K^+^ ratio in *Arabidopsis* roots and increased salt tolerance in *Arabidopsis* transformants (Zhou *et al*., 2015). SbWRKY50 participated in the salt response by controlling ionic homeostasis in *Sorghum bicolor* (sweet sorghum). AtWRKY22 conferred tolerance to submergence by interacting with the ACS7 promoter and activating downstream ethylene signalling (Hsu *et al*., 2013). However, the mechanisms by which upstream factors activate WRKYs and regulate stress resistance‐associated pathways in plants remain to be clarified (Rushton *et al*., 2010).

Our previous study showed that autotetraploid apple plants have higher salt stress tolerance than diploid apple plants (Xue *et al*., 2015). The reason may be related to the former have extra copies of the genes conferring salt resistance to the plant by homologous doubling process. Digital gene expression and microarray analyses revealed significant differences between autotetraploid and diploid apples in terms of *MIR156a* and *MdSPL13‐like* expression (Ma *et al*., 2016), to elucidate the mechanism of miR156/SPL regulating salt stress in apple. Here, we found that *MIR156a* and *MdSPL13* both respond to salt stress but display different expression patterns. We used miR156 as the entry point and investigated the miR156/SPL regulatory network of downstream genes that enhance salt tolerance in apple.

## Results

### The miR156 target *MdSPL13* regulates salt tolerance in apple

**Figure 1:**
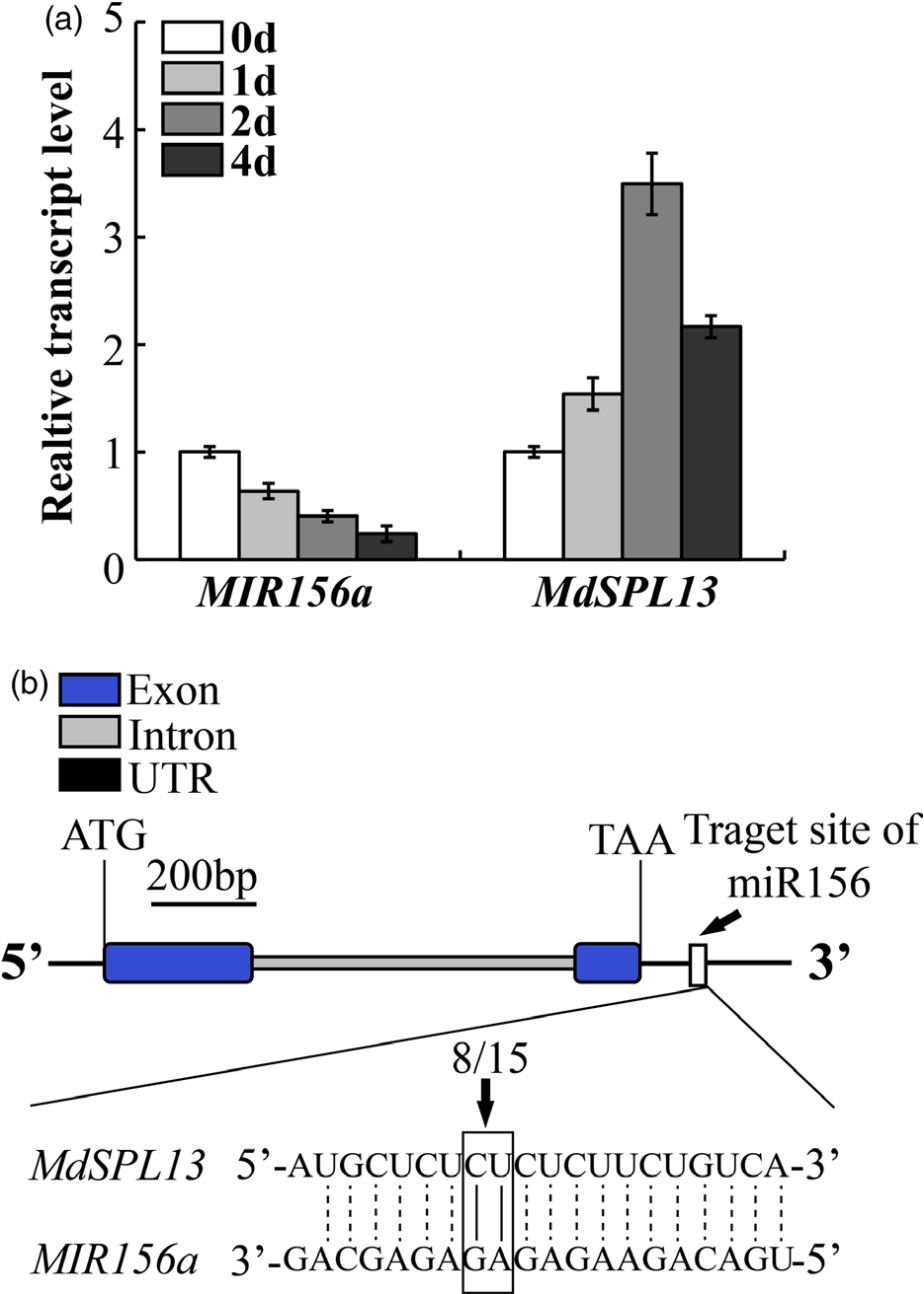
*MdSPL13* is the miR156 target. (a) *MIR156a* and *MdSPL13* expression in ‘Hanfu’ apple under salt stress treatment. (b) *MdSPL13* is the miR156 target. Error bars indicate standard deviation (SD) for three biological replicates. [Colour figure can be viewed at wileyonlinelibrary.com]

‘Hanfu’ apple plants were treated with 150 mm NaCl. The phenotype of apple plants under salt treatment was shown in Figure S1a. The leaves were little withered at 2 day treatment and local necrosis at 4 day. The *MIR156a* expression decreased with time under the salt stress treatment (Figure 1a). In contrast, *MdSPL13* expression increased with time under the salt stress treatment during 0–2 day treatment (Figure 1a). The expression patterns of other 9 *MdSPL* genes, which had the putative miR156 target sites (Li *et al*., 2013 and Zhang *et al*., 2014), under salt tolerance were tested. The data were supplemented in Figure S1b‐j. There were two expression patterns of these 9 *MdSPL* genes under salt treatment during 0–2 day. One expression pattern was first increase then decrease, such as *MdSPL8*, *MdSPL11*, *MdSPL24*, *MdSPL25* and *MdSPL28*. The other was decrease, such as *MdSPL5*, *MdSPL16*, *MdSPL17* and *MdSPL27*. Neither of them was like the expression pattern of *MdSPL13*. We cloned the 3′ UTR sequence of *MdSPL13* from ‘Hanfu’ apple leaves and analysed the relationship between miR156 and *MdSPL13* by RLM‐5′RACE. As shown in Figure 1b, *MdSPL13* had a single miR156 target located on the 3′ UTR and the cleavage frequency was 8/15 (~53%).

### *MIR156a* overexpression decreases salt tolerance in apple

To explore the function of *MIR156a* in apple, we obtained the transgenic *MIR156a* overexpression apple lines MIR156aOE‐6 and MIR156aOE‐8 via *Agrobacterium*‐mediated transformation of ‘GL‐3’ leaves. The *MIR156a* expression levels in the MIR156aOE lines were fourfold‐ sevenfold higher than those in the nontransgenic ‘GL‐3’ plants (Figure S2a). The *MdSPL13* expression levels in the MIR156aOE lines were 37%–42% lower than those in the nontransgenic ‘GL‐3’ plants (Figure S2b).

**Figure 2:**
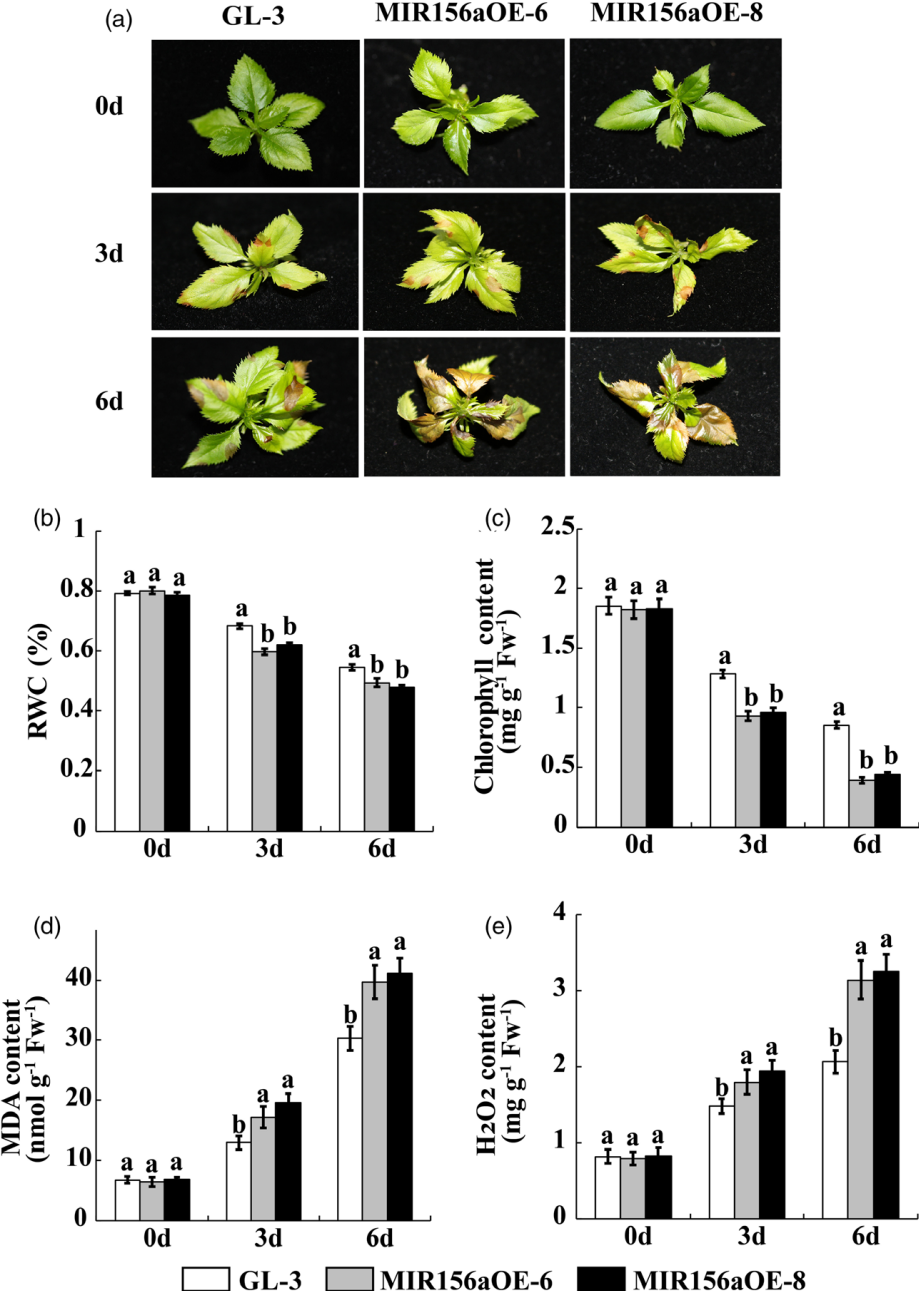
*MIR156a* overexpression decreases salt tolerance in apple. (a) Phenotypic analysis of ‘GL‐3’ and MIR156aOE lines under salt stress treatment. (b‐e) RWC, chlorophyll, MDA and H_2_O_2_ content in ‘GL‐3’ and MIR156OE leaves under salt stress treatment. Error bars indicate standard deviation (SD) for three biological replicates. Different letters indicate significant differences between treatment means (*P* < 0.05; Duncan’s multiple range test). [Colour figure can be viewed at wileyonlinelibrary.com]

The nontransgenic ‘GL‐3’ and the transgenic MIR156aOE lines were treated with 150 mm NaCl and the former served as a control. We observed no obvious phenotype differences between ‘GL‐3’ and the transgenic lines before the salt stress treatment. Leaf injury was detected in the ‘GL‐3’ and MIR156aOE lines after 3 day salt stress treatment. The damage was particularly severe in the MIR156aOE lines. The ‘GL‐3’ plants showed strong salt tolerance whereas nearly all of the MIR156aOE lines became wilted and chlorotic after 6 d salt stress treatment (Figure 2a).

Reactive oxygen species (ROS) are produced in plants subjected to NaCl stress. We used 3,3‐diaminobenzidine (DAB) and nitro blue tetrazolium (NBT) staining to measure the H_2_O_2_ and O_2_^‐1^ levels in the leaves of the apple plants exposed to salt stress. The active oxygen content was higher in MIR156aOE than it was in ‘GL‐3’ (Figure S3).

The foliar relative water content (RWC) reflects the ability of the plant to retain water under stress conditions. Moreover, stress may damage leaves and degrade chlorophyll. Here, both the RWC and the chlorophyll content in ‘GL‐3’ leaves were significantly higher than those in MIR156aOE leaves in response to salt stress (Figure 2b and c). The malondialdehyde (MDA) content indicates the degree of membrane lipid peroxidation which, in turn, reflects the degree of plant injury under stress conditions. MDA accumulation was significantly lower in the ‘GL‐3’ plants than it was in the MIR156aOE plants (Figure 2d). The H_2_O_2_ content in the MIR156aOE leaves was significantly higher than that in the ‘GL‐3’ leaves in response to salt stress (Figure 2e).

### MdSPL13 acts as a transcriptional activator

**Figure 3:**
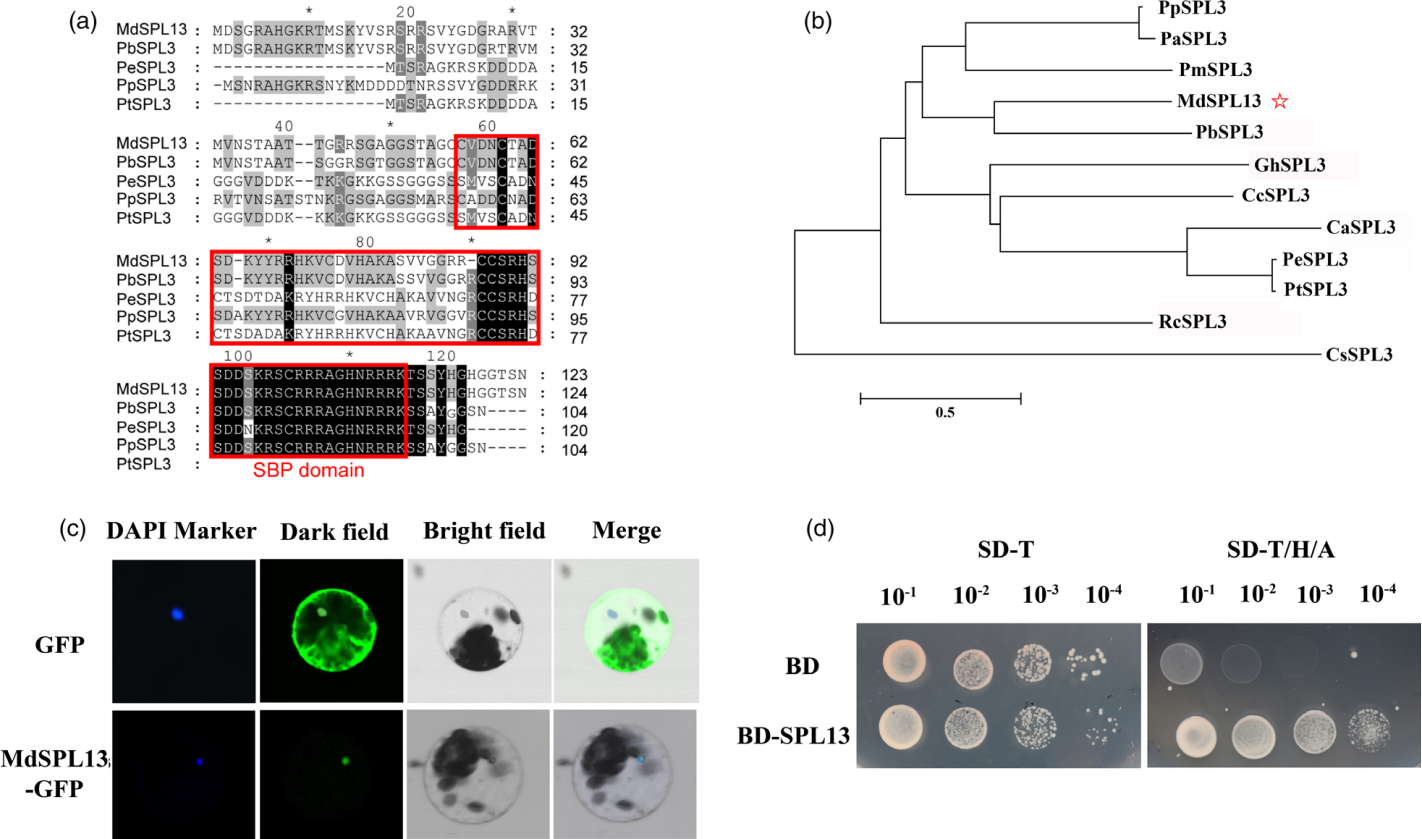
MdSPL13 transcription factor analysis. (a) Amino acid sequence alignment of MdSPL13 and its homologs in various plants. (b) Phylogenetic tree analyses of MdSPL13 and SPL3 family proteins derived from other plant species. (c) Subcellular location of MdSPL13 transcription factor. (d) Transcriptional activity of MdSPL13 protein. [Colour figure can be viewed at wileyonlinelibrary.com]

The coding sequence (CDS) of the gene encoding the MdSPL13 transcription factor (MD10G1291800) is 507 nucleotides long. It encodes a peptide 168 amino acids long with MW = 19 kDa. Sequence alignment and phylogenetic analysis revealed that MdSPL13 has the SBP domain and strong homology with pear (*Pyrus bretschneideri*) which is also a member of the Rosaceae (Figure 3a and b). The spatiotemporal expression analysis revealed that the *MdSPL13* expression levels were higher in the leaves, stems and flowers than they were in the roots and fruits (Figure S2c).

To determine the subcellular location of the MdSPL13 transcription factor, we constructed the p35S::MdSPL13‐eGFP vector and introduced it into *Arabidopsis* protoplasts. The pRI101‐eGFP empty vector served as a control. We observed the MdSPL13‐eGFP fusion protein signal under a confocal microscope. Both the DAPI and green fluorescence signals were clearly evident in the protoplast nuclei. Thus, MdSPL13 is localized to the nucleus (Figure 3c). To examine MdSPL13 protein transcriptional activation, we fused the *MdSPL13* CDS region to the GAL4 DNA‐binding domain and then introduced BD‐MdSPL13 into the Y2H yeast strain. The yeast strain with BD‐MdSPL13 can grow in both SD‐T and SD‐T/H/A culture medium even at the dilution of 10^−4^. At the same time, the yeast strain with BD can grow in SD‐T culture medium but cannot grown in SD‐T/H/A at the dilution of 10^−2^–10^−4^ (Figure 3d). It suggested that MdSPL13 acted as a transcriptional activator.

### *MdSPL13* overexpression improves salt tolerance in apple

To verify the biological function of *MdSPL13* in apple, we generated the transgenic *MdSPL13‐*overexpressing apple lines MdSPL13OE‐2, MdSPL13OE‐5, MdSPL13OE‐8, MdSPL13OE‐11 and MdSPL13OE‐16 via *Agrobacterium*‐mediated ‘GL‐3’ apple transformation. The relative expression levels of *MdSPL13* were eightfold to 14‐fold higher in the MdSPL13OE transgenic lines than they were in the ‘GL‐3’ plants (Figure S2d).

**Figure 4:**
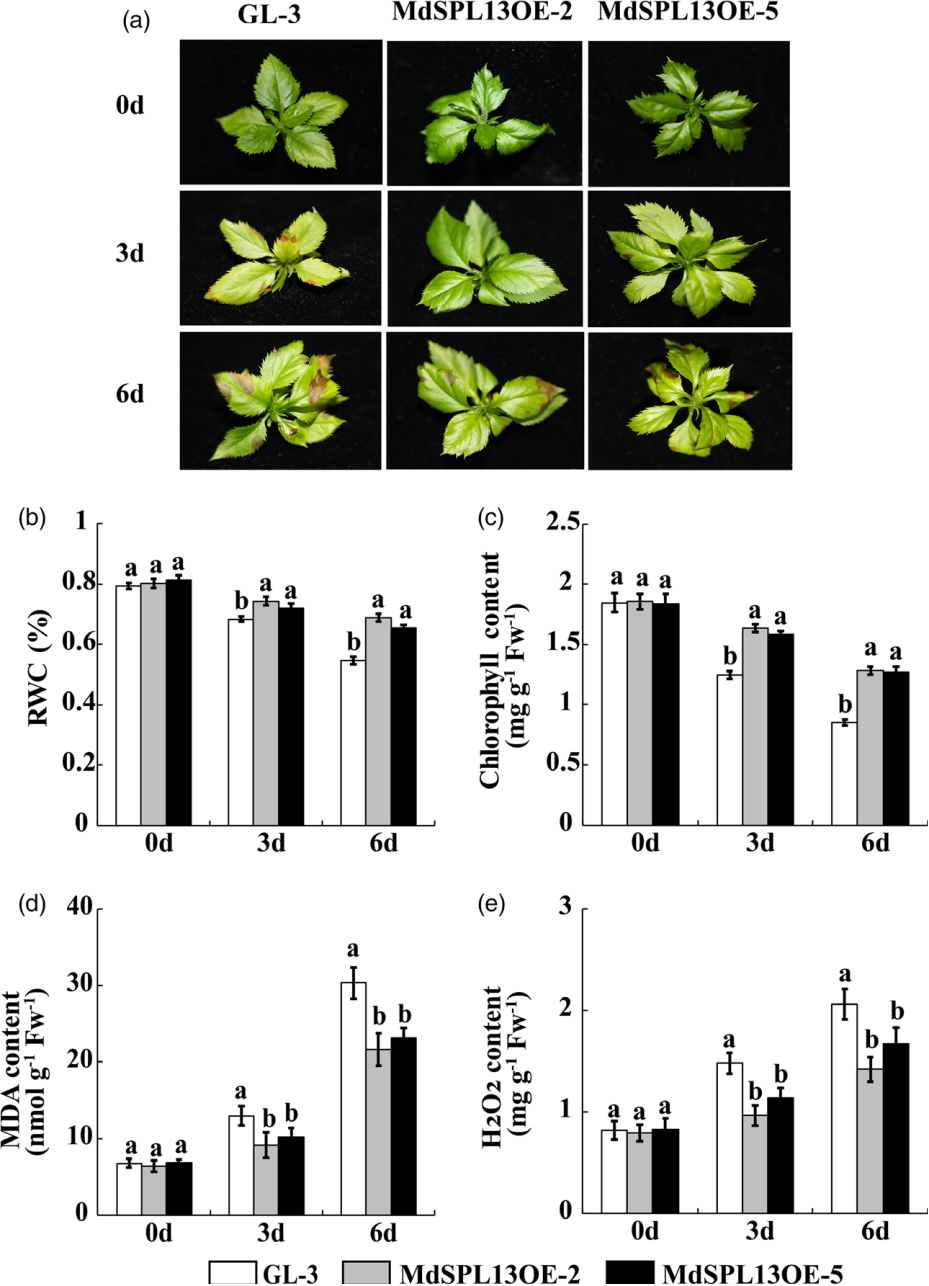
*MdSPL13* overexpression increases salt tolerance in apple. (a) Phenotypes of ‘GL‐3’ and MdSPL13OE lines under salt stress treatment. (b‐e). RWC and chlorophyll, MDA and H_2_O_2_ content in ‘GL‐3’ and MdSPL13OE leaves under salt stress treatment. Error bars indicate standard deviation (SD) for three biological replicates. Different letters indicate significant differences between treatment means (*P* < 0.05; Duncan’s multiple range test). [Colour figure can be viewed at wileyonlinelibrary.com]

The ‘GL‐3’ and MdSPL13OE transgenic lines MdSPL13OE‐2 and MdSPL13OE‐5 were treated with 150 mm NaCl. We observed no distinct phenotypic differences between the ‘GL‐3’ and transgenic plants before the salt stress treatment. After 3‐day salt stress treatment, however, ‘GL‐3’ presented with wilted and chlorotic leaves whereas the MdSPL13OE lines showed no obvious phenotypic alterations in their leaves. After 6‐day salt stress treatment, the MdSPL13OE lines exhibited wilted and chlorotic leaves while those of ‘GL‐3’ were even more severely injured (Figure 4a).

The DAB and NBT staining showed that the active oxygen content was greater in the ‘GL‐3’ leaves than it was in the MdSPL13OE leaves in response to salt stress (Figure S3). The RWC and chlorophyll content in the MdSPL13OE leaves were higher than those in ‘GL‐3’ after 0, 3‐ and 6‐day salt stress durations (Figures 4b and c). MDA accumulation was significantly higher in ‘GL‐3’ than it was in MdSPL13OE in response to salt stress (Figure 4d). The H_2_O_2_ content was significantly higher in ‘GL‐3’ than it was in MdSPL13OE (Figure 4e).

### Transcriptomic analysis reveals differentially expressed genes in both MdSPL13OE and ‘GL‐3’ plants

We conducted a transcriptomic analysis (RNA‐Seq) of MdSPL13OE and ‘GL‐3’ leaves in order to elucidate the MdSPL13 regulatory network. A total of 6094 differentially expressed genes (DEGs) were found. Of these, 1798 were up‐regulated and 4296 were down‐regulated in the MdSPL13OE leaves relative to the ‘GL‐3’ leaves (Figure S4). A gene ontology (GO) enrichment analysis showed that the DEGs were mainly associated with antioxidant, nucleic acid‐binding transcription factor and enzyme regulator activity (Figure S5).

**Figure 5:**
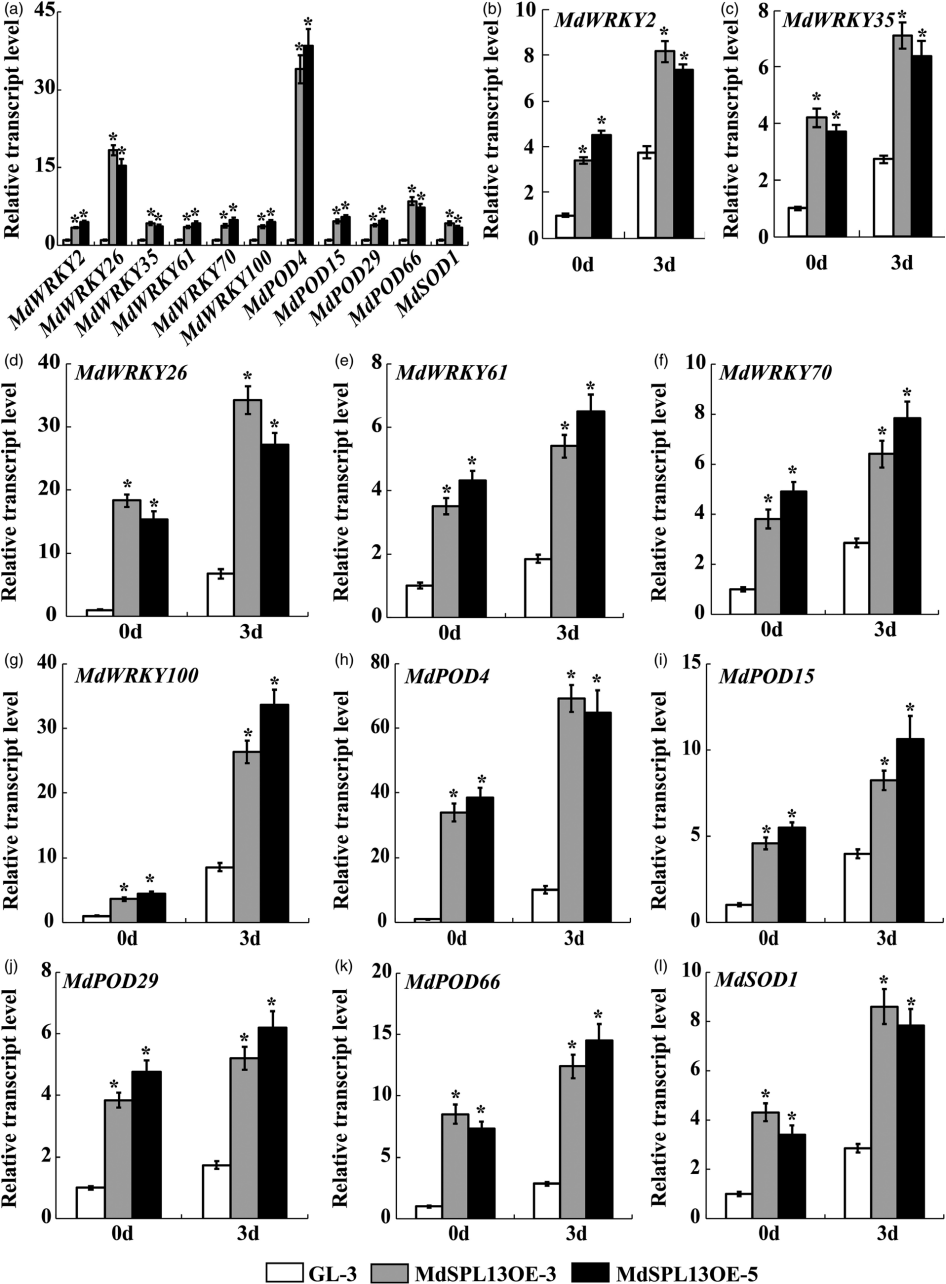
Differential gene expression analyses of ‘GL‐3’ and MdSPL13OE lines. (a) qRT‐PCR analysis of DEGs to verify RNA‐Seq data. (b‐l) DEG expression in MdSPL13OE lines under salt stress treatment. Error bars indicate standard deviation (SD) for three biological replicates. Asterisks indicate significant differences between treatment means (*P* < 0.05; *t*‐test).

To verify the RNA‐Seq data, 11 genes encoding *WRKYs*, *PODs* or *SODs* were selected for qRT‐PCR. Compared with the ‘GL‐3’ plants, the MdSPL13OE plants exhibited significantly up‐regulated *MdWRKY2*, *MdWRKY26*, *MdWRKY35*, *MdWRKY61*,*MdWRKY70*, *MdWRKY100*, *MdSOD1*, *MdPOD4*, *MdPOD15*, *MdPOD29* and *MdPOD66*. These findings were consistent with the RNA‐Seq data and the latter were reliable. All of the above‐mentioned genes had ≥1 GTAC motif (SBP‐binding element) in their promoters (Figure 5a).

The preceding experiments demonstrated that the MdSPL13‐OE lines had superior salt tolerance to the ‘GL‐3’ plants and the MIR156‐OE lines were opposite. The genes associated with this trait we selected from DEGs data were significantly up‐regulated in the MdSPL13‐OE lines and down‐regulated in MIR156‐OE lines relative to the ‘GL‐3’ plants. *MdWRKY26*, *MdWRKY100* and *MdPOD4* were the most significantly different expression genes in response to the salt stress treatment in both MdSPL13‐OE lines and MIR156‐OE lines with different expression pattern (Figures 5b‐l; Figure S6a‐k). Therefore, we selected *MdWRKY26*, *MdWRKY100* and *MdPOD4* for the subsequent assays.

### MdSPL13 binds and activates the *MdWRKY100* promoter

**Figure 6:**
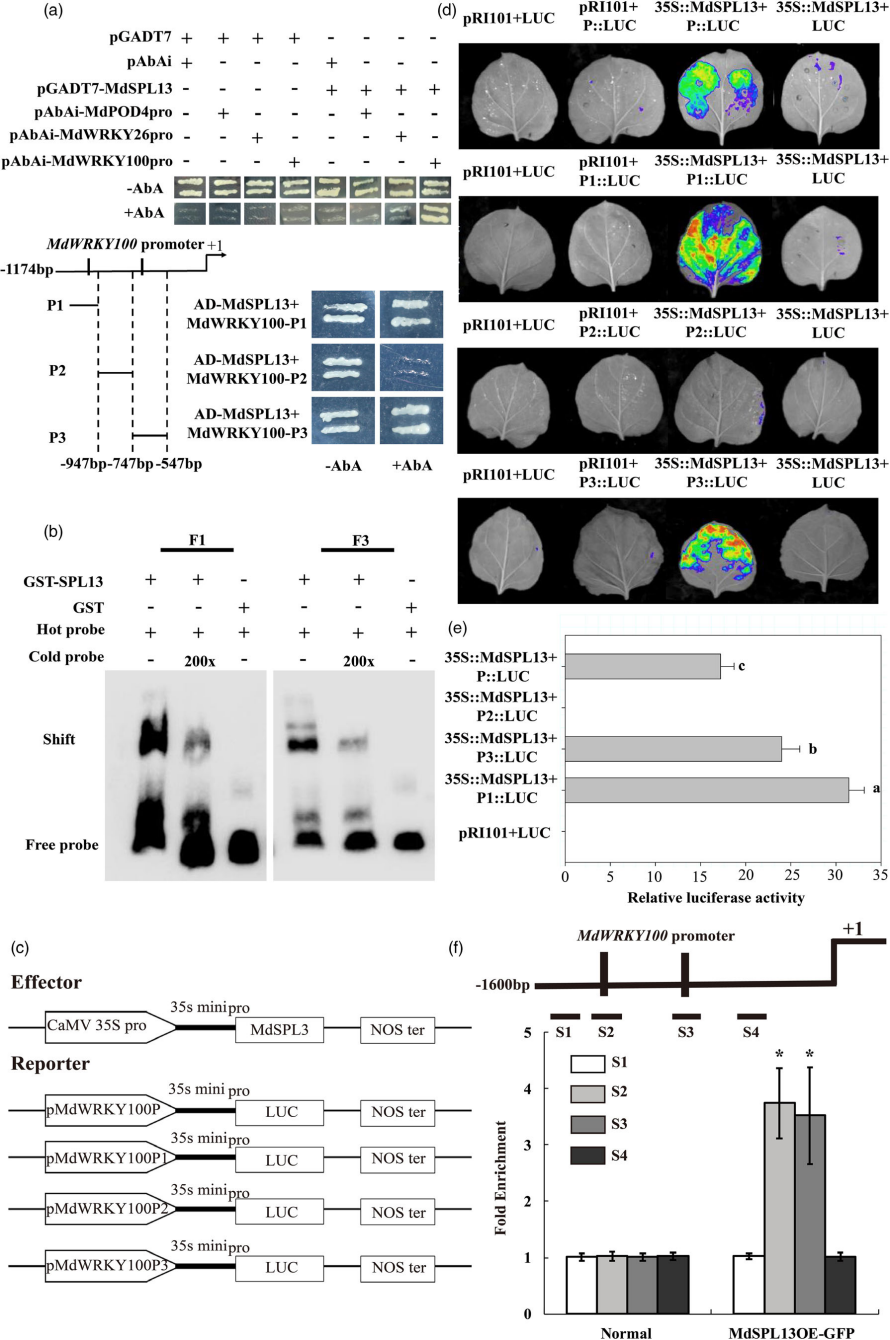
MdSPL13 is a transcriptional activator of *MdWRKY100*. (a) Yeast one‐hybrid assay of MdSPL13 binding to the *MdWRKY100* promoter. The *MdWRKY100* promoter sequence was divided into P1, P2, and P3. Aureobasidin A (AbA), an inhibitor of yeast call growth, was used as a screening marker. The concentration of AbA was 150 ng/mL for *MdPOD4*, *MdWRKY26* and *MdWRKY100* promoter fragments and 100 ng/mL for P1, P2 and P3. (b) EMSA was performed using probes specific to the *MdWRKY100* promoter F1 and F3 fragments. Binding competition was tested using 200 × competitive probes. GST was the negative control. (c) Schematic diagrams of the effector and reporter constructs used in the tobacco transient expression assay. (d) Luciferase activity assay showed that MdSPL13 binds the P1 and P3 fragments of the *MdWRKY100* promoter. (e) Analysis of luciferase activity. Different letters indicate significant differences between treatment means (*P* < 0.05; Duncan’s multiple range test). (f) ChIP assay of MdSPL13 binding to the *MdWRKY100* promoter S2 and S3 fragments. Error bars indicate standard deviation (SD) for three biological replicates. Asterisks indicate significant differences between treatment means (*P* < 0.05; *t*‐test). [Colour figure can be viewed at wileyonlinelibrary.com]

In this study, *MdWRKY26*, *MdWRKY100* and *MdPOD4* were selected for the yeast one‐hybrid assay in order to determine their interactions with MdSPL13 transcription factors. The MdSPL13 protein could only directly bind the *MdWRKY100* promoter (Figure 6a). Figures 6a show two GTAC motifs on the *MdWRKY100* promoter. The *MdWRKY100* promoter fragment was divided into three parts, namely, P1, P2 and P3, and assayed with the MdSPL13 protein. MdSPL13 bound the P1 and P3 fragments which include the GTAC‐binding sites of the *MdWRKY100* promoter. However, MdSPL13 did not bind the P2 fragment as it lacked the GTAC‐binding sites.

Figure 6b shows that according to the EMSA, the MdSPL13 protein bound F1 and F3 biotin‐labelled probes designed according to the P1 and P3 fragments containing the GTAC motif. An unlabelled competitive probe competed with the biotin‐labelled probe for MdSPL13 binding. MdSPL13 can bind the GTAC motif‐bearing F1 and F3 fragments in the WRKY100 promoter.

We introduced the P1 and P3 fragments into a pRI‐LUC vector containing the luciferase reporter gene (Figure 6c). We injected the pMdWRKY100‐LUC product into tobacco leaves along with the effector p35S::MdSPL13. We measured fluorescence to detect luciferase activity in the tobacco leaves after 72 h. MdSPL13 activated *MdWRKY100* transcription (Figure 6d and e). According to the ChIP‐qPCR (Figure 6f), MdSPL13 could bind the S2 and S3 fragments including the GTAC‐binding sites of the *MdWRKY100* promoter.

### *MdWRKY100* regulates salt stress tolerance in apple

**Figure 7:**
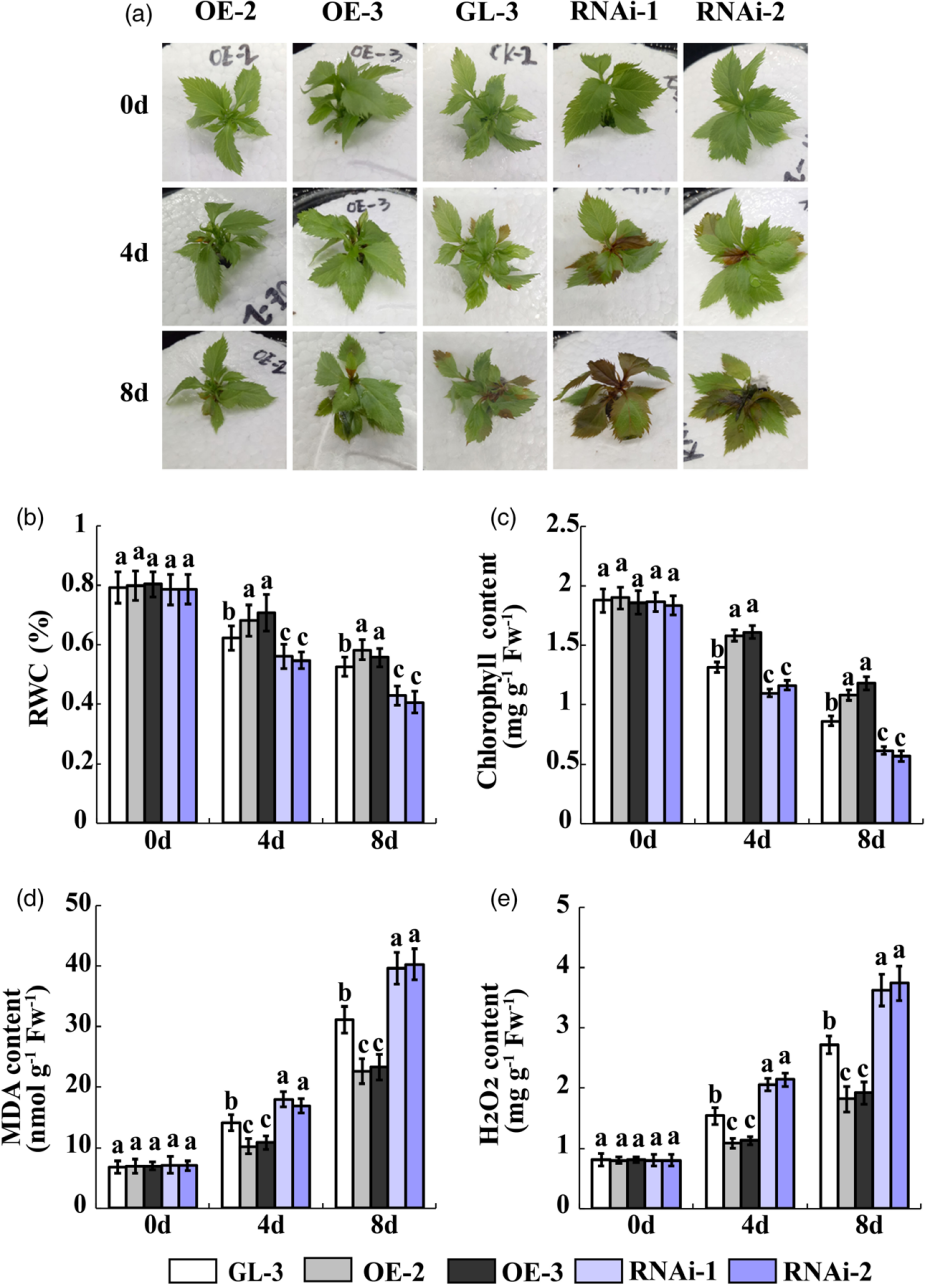
*MdWRKY100* gene regulates salt tolerance in apple. (a) Phenotypes of MdWRKY100OE, ‘GL‐3’ and MdWRKY100RNAi lines under salt stress treatment. (b‐e) RWC and chlorophyll, MDA and H_2_O_2_ content in MdWRKY100OE, ‘GL‐3’ and MdWRKY100RNAi leaves under salt stress treatment. Error bars indicate standard deviation (SD) for three biological replicates. Different letters indicate significant differences between treatment means (*P* < 0.05; Duncan’s multiple range test). [Colour figure can be viewed at wileyonlinelibrary.com]

In our previous study, we obtained *MdWRKY100* transgenic lines and discovered that *MdWRKY100* overexpressing lines strongly resist *Colletotrichum gloeosporidoides* infection (Zhang *et al*., 2019). Here, we selected ‘GL‐3’, the MdWRKY100OE (MdWRKY100OE‐2 and MdWRKY100OE‐3) lines and the MdWRKY100RNAi (MdWRKY100RNAi‐1 and MdWRKY100RNAi‐2) lines to examine the salt tolerance function of *MdWRKY100* in apple. The performance of the MdWRKY100OE lines was superior to that of ‘GL‐3’ under salt stress and the MdWRKY100RNAi lines displayed the poorest salt tolerance performance (Figure 7a). The RWC and chlorophyll content in the MdWRKY100OE lines were higher than those in the ‘GL‐3’ and MdWRKY100RNAi lines. The MdWRKY100RNAi lines exhibited the lowest RWC and chlorophyll content (Figure 7b and c). The H_2_O_2_ and MDA levels were consistently higher in the MdWRKY100RNAi lines than they were in the ‘GL‐3’ and MdWRKY100OE lines. The MdWRKY100OE lines presented with the lowest H_2_O_2_ and MDA accumulation (Figure 7d and e). Phenotypes of *MIR156a*, *MdSPL13* and *MdWRKY100* overexpression plants and *MdWRKY100* RNAi plants and ‘GL‐3’ under salt treatment at one experiment were shown in Figure S7.

## Discussion

The miR156/SPL module regulates leaf development, fruit ripening, vegetative and reproductive stage transitions, tillering and branching, panicle and tassel architecture, fertility, and abiotic stress response of plant (Cui *et al*., 2020; Fu *et al*., 2012; Gao *et al*., 2018; Gou *et al*., 2011; Long *et al*., 2018; Tian *et al*., 2014; Zhang *et al*., 2015). A few reports investigated the roles of the miR156/SPL module in salt tolerance by integrating miRNA‐Seq and RNA‐Seq data (Wang *et al*., 2019a). The salt tolerance functions of certain *SPL* genes were confirmed by heterologous expression in *Arabidopsis* (Hou *et al*., 2018; Ning *et al*., 2017). One comprehensive study on *Arabidopsis* demonstrated that the miR156/SPL module regulates DIHYDROFLAVONOL‐4‐REDUCTASE (DFR) and enables the plant to adapt to salt stress via anthocyanin metabolism (Cui *et al*., 2014). Overall, the mechanism by which the miR156/SPL module regulates plant salt tolerance is poorly understood. Our previous studies showed that salt‐sensitive diploid and salt‐tolerant autotetraploid apple significantly differed in terms of their *MIR156a* and *MdSPL13* expression levels (Ma *et al*., 2016; Xue *et al*., 2015). Here, we identified for the first time that *MdSPL13* is the target gene of miR156 in apple (Figure 1b). Stable genetic transformation technology revealed that the miR156/SPL13 module responds to salt stress in apple (Figures 2 and 4). WRKY TFs regulate ABA synthesis, ROS clearance, ionic balance and ethylene response pathways in order to defend plants against salt stress (Chen *et al*., 2012; Ding *et al*., 2015; Meng *et al*., 2018; Wei *et al*., 2008). The mechanism by which salt stress activates WRKYs and modulates stress tolerance‐related pathways in plants is a subject of growing interest (Rushton *et al*., 2010). The MAPK pathway phosphorylated WRKY8 transcription factors in response to salt stress in *Nicotiana benthamiana* (Ishihama *et al*., 2011). *Arabidopsis* RPD3‐like histone deacetylase HDA9 repressed the salt stress tolerance response by regulating WRKY53 DNA binding and transcriptional activity (Zheng *et al*., 2019). Nevertheless, little is known about the upstream regulatory mechanism of WRKY. In the present study, we discovered that MdSPL13 binds the *MdWRKY100* promoter region and positively regulates *MdWRKY100* expression (Figures 3, 6 and 7). Hence, this research disclosed a novel mechanism wherein the miR156/SPL module regulates salt stress tolerance in apple by activating *MdWRKY100*.

We also demonstrated that salt stress treatment down‐regulated *MIR156a* and up‐regulated the expression of *MdSPL13* except after 4 day (Figure 1a). The reason is after 4‐day salt treatment the plants were serious damage with leaves wilting and local necrosis. Part of the cell death and RNA degradation make both *MIR156a* and *MdSPL13* were down‐regulated at that time (Figure S1a). The miR156 expression pattern observed in apple under salt stress was similar to that reported for maize (*Zea mays*) (Ding *et al*., 2009) but substantially different from those detected in *Arabidopsis*, sugarcane and rice (*Oryza sativa*; Gentile *et al*., 2015; Khraiwesh *et al*., 2012; Liu *et al*., 2008). It is not understood why *Arabidopsis* and apple differ in terms of their *miR156*/*SPL* module expression patterns under salt stress. Differences between woody and herbaceous plants in terms of their life cycles might partially explain this phenomenon. The life cycles of annual herbaceous plants are shorter than those of perennial woody plants. When plants are challenged by salt stress, they allocate energy towards defence mechanisms and the miR156 level increases. Moreover, expression of the SPLs regulated by miR156 is inhibited. These responses prolong the juvenile phase of the plant and enable it to withstand unfavourable environmental conditions. Conversely, when ambient conditions improve, the miR156 level decreases and flowering accelerates (Cui *et al*., 2014). Unlike *Arabidopsis* and other herbaceous plants, the woody perennial apple has a very long juvenile stage (3–5 year). Further, the relationship between the changes in its developmental stages and its salt stress tolerance may be more complex than that of herbaceous plants. When apple is subjected to salt stress, it down‐regulates miR156 in order to up‐regulate the downstream target SPLs. Certain SPLs enhance lateral root elongation and biomass accumulation to counteract the increase in energy consumption triggered by salt shock (Fu *et al*., 2012). Other SPLs target downstream regulatory genes that participate in the physiological and metabolic processes activated in response to salt stress (Wang *et al*., 2019a). Moreover, relative differences in salt stress treatment time and tissue type may account for the observed differences in miR156 expression patterns under salt stress. MiR156 expression at 0.5 h and 2.5 h salt stress was increased in *Arabidopsis* leaves but decreased in maize roots (Ding *et al*., 2009; Liu *et al*., 2008).

Compare to adult tree, our study on tissue culture plants cannot completely explain the mechanism of miR156/SPL module regulates salt tolerance in apple. In further studies, we will transplant this transformation lines in the field on soil and validate them in terms of their fruit yield and quality, nutrient assimilation and water use under salt stress.

Therefore, our study demonstrated that the miR156/SPL module regulates salt tolerance in apple by up‐regulating MdWRKY100. It was the first to identify and confirm the miRNA network response to salt tolerance in apple by standard molecular methods. As apple is closely related to numerous other commercially important fruit trees and ornamental crops in the Rosaceae family, our discoveries may also be applied to reveal molecular mechanism of salt tolerance into those plant species as well.

## Materials and methods

### Plant materials

Tissue cultures of the apple genotypes ‘Hanfu’ and ‘GL‐3’ were used in the salt stress treatments and genetic transformations. The ‘Hanfu’ and ‘GL‐3’ tissue cultures were prepared according to the method of Xue *et al*. (2015). The plants were grown under long‐day conditions (light:dark = 14 h:10 h) at 25 °C. *Arabidopsis* was used for subcellular localization. *Nicotiana benthamiana* was used for transient gene expression. They were grown under short‐day conditions (light:dark = 12 h:12 h) at 23 °C. The MdWRKY100OE and MdWRKY100RNAi apple lines were generated according to the method of Zhang *et al*. (2019). Roots, stems, leaves, flowers and fruits were sampled from 7‐year ‘Hanfu’ apple plants grown under natural conditions in a field at Shenyang Agricultural University.

### Vector construction and genetic transformation

The *mdm‐MIR156a* sequence was cloned from ‘Hanfu’ DNA, and the full‐length CDS region of *MdSPL13* (MD10G1291800) was cloned from ‘Hanfu’ cDNA. Both sequences were inserted into the pRI101‐AN vector with the CaMV 35S promoter. The genes were transformed according to an established protocol (Dai *et al*., 2013). All of the primers used in vector construction are listed in Table S1.

### Confirmation of the mdm‐miR156 target by RLM‐5’RACE

The 5’‐rapid amplification of cDNA ends (5′‐RACE) was used to identify *MdSPL13* as a target gene of mdm‐miR156. The 5′‐RACE was performed with a First Choice RLM‐RACE kit (Ambion, Burlington, ON, Canada) according to the manufacturer’s instructions. The PCR products were cloned and introduced into a pMD‐18T vector. All positive clones were confirmed by PCR. Fifteen clones were sequenced with pMD‐18T vector universal primers. All of the primers used in 5′‐RACE are listed in Table S2.

### Subcellular localization

The full‐length CDS of *MdSPL13* minus the stop codon was cloned from ‘Hanfu’ cDNA (Table S1) and inserted into the pRI101‐eGFP vector to construct the p35S::MdSPL13‐eGFP expression vector. The p35S::MdSPL13‐eGFP plasmid was introduced into *Arabidopsis* protoplasts to observe the GFP signal after 12‐h darkness. The p35S::eGFP vector was the control. The GFP signal was observed under a Leica confocal laser‐scanning microscope (TCS SP8‐SE; Leica, Wetzlar, Germany). The methods used were previously described (Zhang *et al*., 2019).

### Transcriptional activation analysis

The *MdSPL13* CDS region was inserted into the pGBKT7 vector containing the DNA‐binding region of GAL4. The product was named BD‐MdSPL13 and transferred to the Y2H strain. The pGBKT7 vector was the control. To examine transcriptional MdSPL13 activation, the Y2H strains were transformed on SD/‐Trp medium and then selected on SD/‐Trp‐His‐Ade medium. The primers used are listed in Table S1. The methods used were previously described (Zhang *et al*., 2019).

### RNA extraction and quantitative real‐time PCR analysis

Total RNA was isolated from leaves by an established method (Zhang *et al*., 2019). The cDNA synthesis and quantitative real‐time PCR (qRT‐PCR) were performed according to established protocols (Zhang *et al*., 2019). The cDNA synthesis and qRT‐PCR of mdm‐miR156 were performed as previously described (Jia *et al*., 2017). An endogenous apple EF‐1α gene (Accession No. DQ341381) was used for normalization. The RNA extracted from each plant served as a single biological replicate for RT‐PCR and qRT‐PCR. There were three biological replicates in total. Each line of infected apple callus represents a single biological replicate. Three lines of separately infected apple calli were used in this experiment. The primers used in the qRT‐PCR are listed in Table S3.

### Salt stress treatment

Apple tissue culture plants aged 1 month were transferred to a subculture medium containing 150 mm NaCl. After 0, 1, 2 and 4 days of this stress treatment, ‘HF’ apple plants were photographed and used in the subsequent experiments and assays. After 0, 3 and 6 days of this stress treatment, the MiR156aOE lines and MdSPL13OE lines were photographed and used in the subsequent experiments and assays. After 0, 4 and 8 days of this stress treatment, the MdWRKY100OE lines were photographed and used in the subsequent experiments and assays. All of these plants were treated by salt at one experiment. The salt stress treatments were repeated thrice. Three plants were used per replicate.

Leaves of the apple tissue culture plants that had been subjected to above salt stress were collected to measure RWC and chlorophyll, MDA and H_2_O_2_ content. The foliar RWC and chlorophyll and H_2_O_2_ content were measured according to previously reported methods (Liu *et al*., 2011; Velikova *et al*., 2000) The foliar MDA content was determined by the thiobarbituric acid (TBA) test (Cao *et al*., 2013). Each treatment was repeated thrice. Three plants were used per replicate.

### Nitro blue tetrazolium (NBT) and diaminobenzidine (DAB) staining

Leaves of the apple tissue culture plants were subjected to salt stress for different numbers of days and stained with DAB and NBT dye solutions according to previously reported methods (Kumar *et al*., 2014). The DAB and NBT stainings were repeated thrice. Three leaves from each plant were used per replicate.

### Analysis of RNA‐Seq data

RNA‐Seq was performed on ‘GL‐3’ and MdSPL13OE transgenic plants in order to screen for downstream genes affected by the MdSPL13 transcription factor. A DEG subset was selected for qRT‐PCR to verify the reliability of the RNA‐Seq data. All of the genes that were significantly differentially expressed (*P* < 0.05, *t*‐test) were used as inputs for gene ontology (GO) enrichment analysis. RNA‐Seq was repeated for three lines. Three plants per line comprised one replicate. The primers used in the qRT‐PCR are listed in Table S3.

### Yeast one‐hybrid assay

The full‐length *MdSPL13* CDS region was cloned and ligated into a pGADT7 vector. The *MdWRKY26*,*MdWRKY100* and *MdPOD4* promoter fragments were cloned and the gene promoters were ligated into a pAbAi vector. The primers used for the yeast one‐hybrid assays are listed in Table S4. The assay was conducted according to previously reported methods (Li *et al*., 2016). There were two GTAC elements in the *MdWRKY100* promoter sequence. This fragment was then divided into three parts, namely P1, P2 and P3. P1 and P3 included GTAC domain elements whereas P2 did not. Hence, the latter (P2) served as the negative control. Aureobasidin A (AbA), an inhibitor of yeast call growth, was used as a screening marker. The concentration of AbA was 150 ng/mL for *MdPOD4*, *MdWRKY26* and *MdWRKY100* promoter fragments and 100 ng/mL for P1, P2 and P3 fragments.

### Electrophoretic mobility shift assay (EMSA)

The MdSPL13‐GST protein was produced and purified according to the methods of Wang *et al*. (2019b). The F1 and F3 fragments in the *MdWRKY100* promoter included GTAC elements. F1 and F3 were designed as probes according to the method recommended by the manufacturer of the Beyotime biotin probe labelling kit (Beyotime, Shanghai, China). The unlabelled sequence served as a competitor. The EMSA was performed according to the method of Wang *et al*. (2019b). The primers used for the EMSA are listed in Table S4.

### Transcriptional activity analysis

The pRI‐MdSPL13 vector was constructed and defined as an effector. A 2000‐bp fragment containing the *MdWRKY100* promoter binding site region was inserted into the pRI‐mini35S‐LUC vector and defined as a reporter. The pRI‐mini35S‐LUC vector served as a control (Table S4). The transcriptional activity analysis was conducted according to the method of Chen *et al*. (2019).

### Chromatin immunoprecipitation (ChIP) assay

According to the method of Chen *et al*. (2019), a p35S::MdSPL13‐eGFP plasmid was introduced into EHA105 *Agrobacterium* to transform the ‘Ourin’ callus previously subcultured in MS medium. The WRKY100 promoter was divided into four parts, namely, S1, S2, S3 and S4. S2 and S3 contained GTAC domain elements while S1 and S4 did not. The latter two served as negative controls. The ‘Ourin’ callus was the normal. The ChIP assay was run according to the method of Yue *et al*. (2020). Every individual apple callus infection constituted a single biological replicate. Three biological replicates were analysed. All of the primers used in the ChIP assay are listed in Table S4.

### Statistical analysis

DPS v. 7.05 was used for data analysis. After ANOVA, Duncan’s test and Student’s *t*‐test were used to identify significant differences among treatment means. A phylogenetic tree was plotted with MEGA5.1. Bootstrap values were derived from 1000 replicate runs.

## Accession Number

Sequence data from this article can be found in GENOME DATABASE FOR ROSACERE (http://www.rosaceae.org) and GenBank/EMBL libraries. MdSPL13 (MDP0000323003) (*Malus domestica*), MdSPL5 (MDP0000263766) (*Malus domestica*), MdSPL8 (MDP0000262141) (*Malus domestica*), MdSPL11 (MDP0000170630) (*Malus domestica*), MdSPL16 (MDP0000171877) (*Malus domestica*), MdSPL17 (MDP0000155354) (*Malus domestica*), MdSPL24 (MDP0000210138) (*Malus domestica*), MdSPL25 (MDP0000589558) (*Malus domestica*), MdSPL27 (MDP*0000193702)* (*Malus domestica*), MdSPL28 (MDP0000249364) (*Malus domestica*), PbSPL3 (NO. XP_009352877.1) (*Pyrus bretschneideri*), PeSPL3 (NO. XP_011043410.1) (*Populus euphratica*), PtSPL3 (NO. XP_006377387.2) (*Populus trichocarpa*), CcSPL3 (NO. XP_006449372.1) (*Citrus clementina*), PpSPL3 (NO. XP_007212177.1) (*Prunus persica*), PmSPL3 (NO. XP_008225122.1) (*Prunus mume*), CsSPL3 (NO. XP_006467766.3) (*Citrus sinensis*), CaSPL3 (NO. XP_004485802.1) (*Cicer arietinum*), PaSPL3 (NO. XP_021825845.1) (*Prunus avium*), GhSPLSPL3 (NO. XP_016711047.1) (*Gossypium hirsutum*), RcSPL3 (NO. XP_024199161.1) (*Rosa chinensis*), FvSPL3 (NO. XP_004293885.1) (*Fragaria vesca*), PvSPL3 (NO. XP_031278960.1) (*Pistacia vera*), NtSPL3 (NO. XP_016515187.1) (*Nicotiana tabacum*), DzSPL3 (NO. XP_022777344.1) (*Durio zibethinus*), MiSPL3 (NO. QCC72916.1) (*Mangifera indica*), JrSPL3 (NO. XP_018850782.1) (*Juglans regia*) and BpSPL3 (NO. AXB72467.1) (*Betula platyphylla*).

## Conflict of interest statement

The authors have no conflict of interest to declare.

## Author contributions

YM and ZZ designed the experiments. YM and HX wrote the manuscript. HX generated the *MdSPL13* and *mdm‐MIR156a* apple transformants. YZ generated the *MdWRKY100* apple transformants. SY performed the transcriptional activation analysis. FZ, QJ, PY and FW performed the yeast one‐hybrid, EMSA, LUC and ChIP assays. LL and PH performed all other experiments. YM and HX contributed equally to this work.

## Supporting information


**Figure S1** The ‘Hanfu’ apple plants under salt tolerance.
**Figure S2** *MIR156a* and *MdSPL13* expression in ‘GL‐3’ and transgenic apple plants.
**Figure S3** NBT and DAB staining of MdSPL13OE, ‘GL‐3’ and MIR156aOE transgenic plants under salt stress for 0, 3 and 6 days.
**Figure S4** Volcano plot of DEGs identified by RNA‐Seq.
**Figure S5** GO enrichment map of DEGs identified by RNA‐Seq.
**Figure S6** Differential gene expression analyses of ‘GL‐3’ and MIR156aOE lines under salt stress treatment.
**Figure S7** Phenotypes of ‘GL‐3’, MIR156aOE, MdSPL13OE, MdWRKY100OE and MdWRKY100RNAi plants under salt treatment, scale bars = 1 cm.
**Table S1** Primer sequences used for cloning *MIR156a* and *MdSPL13*.
**Table S2** Primer sequences of RLM‐5’RACE.
**Table S3** Primer sequences of qRT‐PCR analysis.
**Table S4** Primer sequences used for cloning promoters.
