# Supplementary material for: The miR156/SPL module regulates apple salt stress tolerance by activating MdWRKY100 expression
Source: Plant Biotechnol J. 2020 Sep 4;19(2):311–23. doi: 10.1111/pbi.13464 (PMC7868983; doi:10.1111/pbi.13464)
Supplement: Supplementary file 1 — Figure S1 The ‘Hanfu’ apple plants under salt tolerance. Figure S2 MIR156a and MdSPL13 expression in ‘GL‐3’ and transgenic apple plants. Figure S3 NBT and DAB staining of MdSPL13OE, ‘GL‐3’ and MIR156aOE transgenic plants under salt stress for 0, 3 and 6 days. Figure S4 Volcano plot of DEGs identified by RNA‐Seq. Figure S5 GO enrichment map of DEGs identified by RNA‐Seq. Figure S6 Differential gene expression analyses of ‘GL‐3’ and MIR156aOE lines under salt stress treatment. Figure S7 Phenotypes of ‘GL‐3’, MIR156aOE, MdSPL13OE, MdWRKY100OE and MdWRKY100RNAi plants under salt treatment, scale bars = 1 cm. Table S1 Primer sequences used for cloning MIR156a and MdSPL13. Table S2 Primer sequences of RLM‐5’RACE. Table S3 Primer sequences of qRT‐PCR analysis. Table S4 Primer sequences used for cloning promoters. [file PBI-19-311-s001.doc]

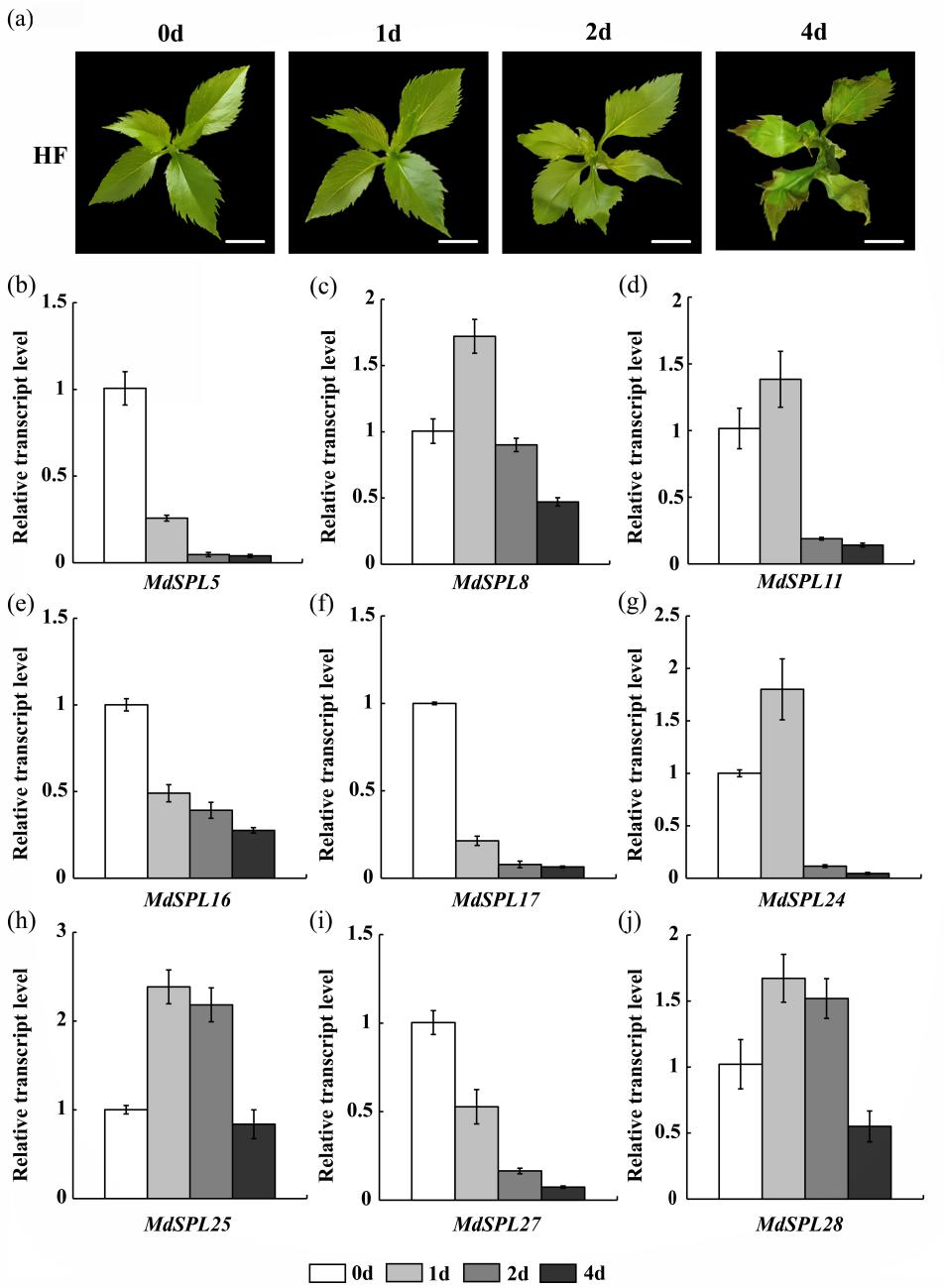


**Fig. S1** The ‘Hanfu’ apple plants under salt tolerance. (a) Phenotypes of ‘Hanfu’ apple plants under salt tolerance after 0, 1, 2, 4 d, scale bars = 1 cm. (b-j) Different *MdSPL* genes expression analyses under salt tolerance after 0, 1, 2, 4 d*.*


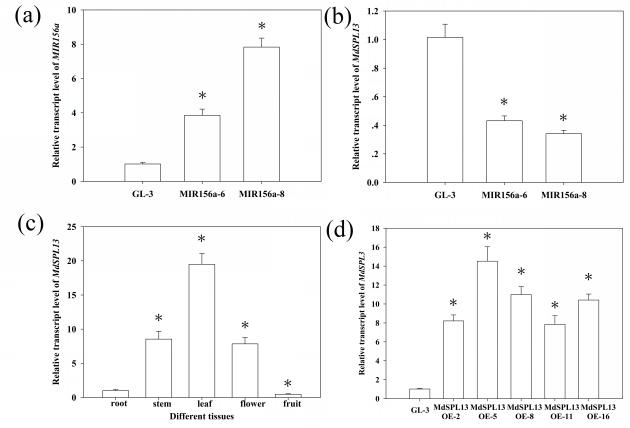


**Fig. S2** *MIR156a* and *MdSPL13* expression in ‘GL-3’ and transgenic apple plants. (a) Gene expression of *MIR156a* in MIR156aOE lines. (b) *MdSPL13* expression in MIR156aOE lines. (c) *MdSPL13* expression in various tissues of ‘Hanfu’ apple. (d) *MdSPL13* expression in MdSPL13OE lines. Error bars indicate standard deviation (SD) for three biological replicates. Asterisk indicates significant differences between treatment means (*P* < 0.05; *t*-test).


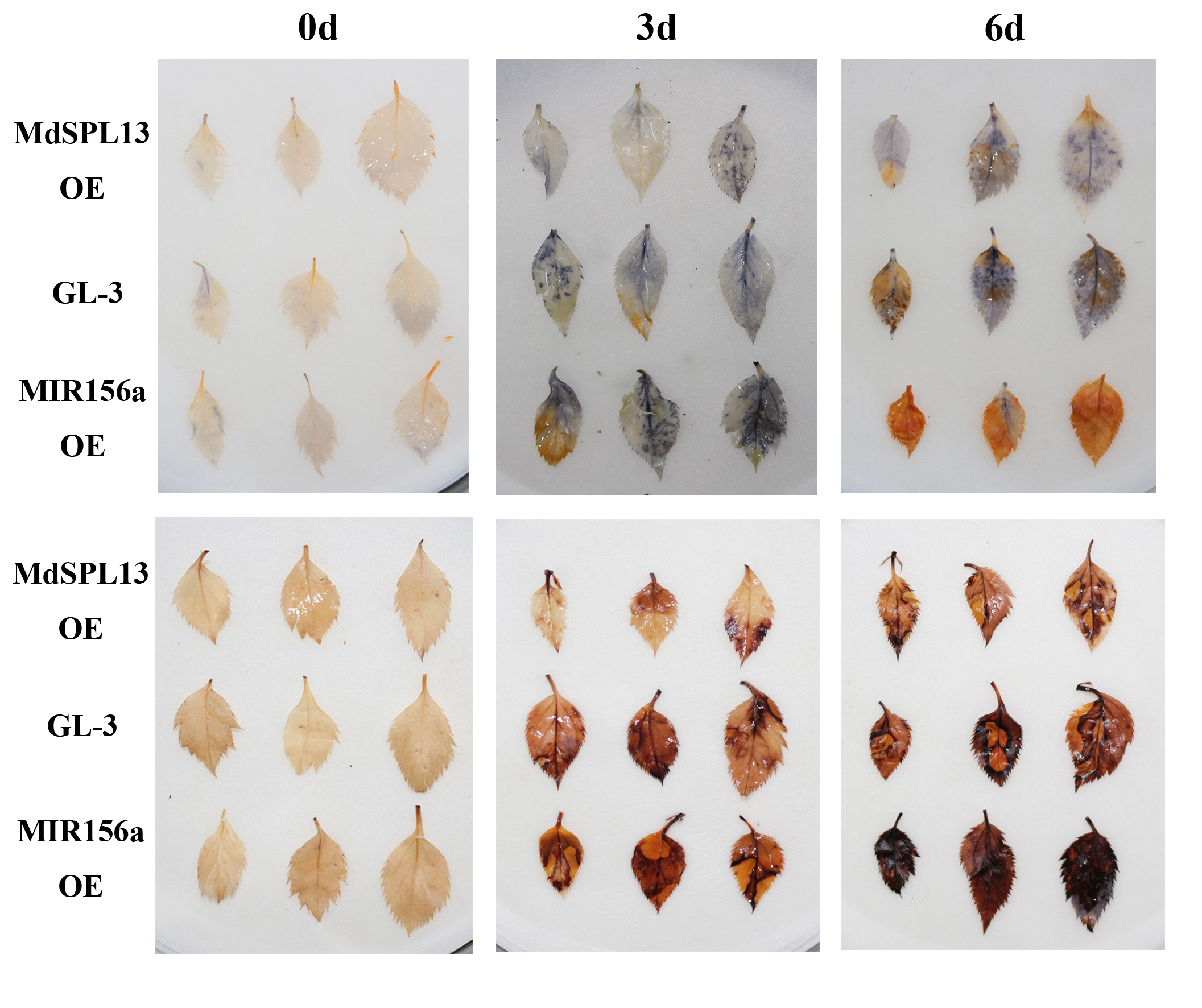


**Fig. S3** NBT and DAB staining of ‘GL-3’ and MdSPL13OE and MIR156aOE transgenic lines under salt stress for 0 d, 3 d, and 6 d.


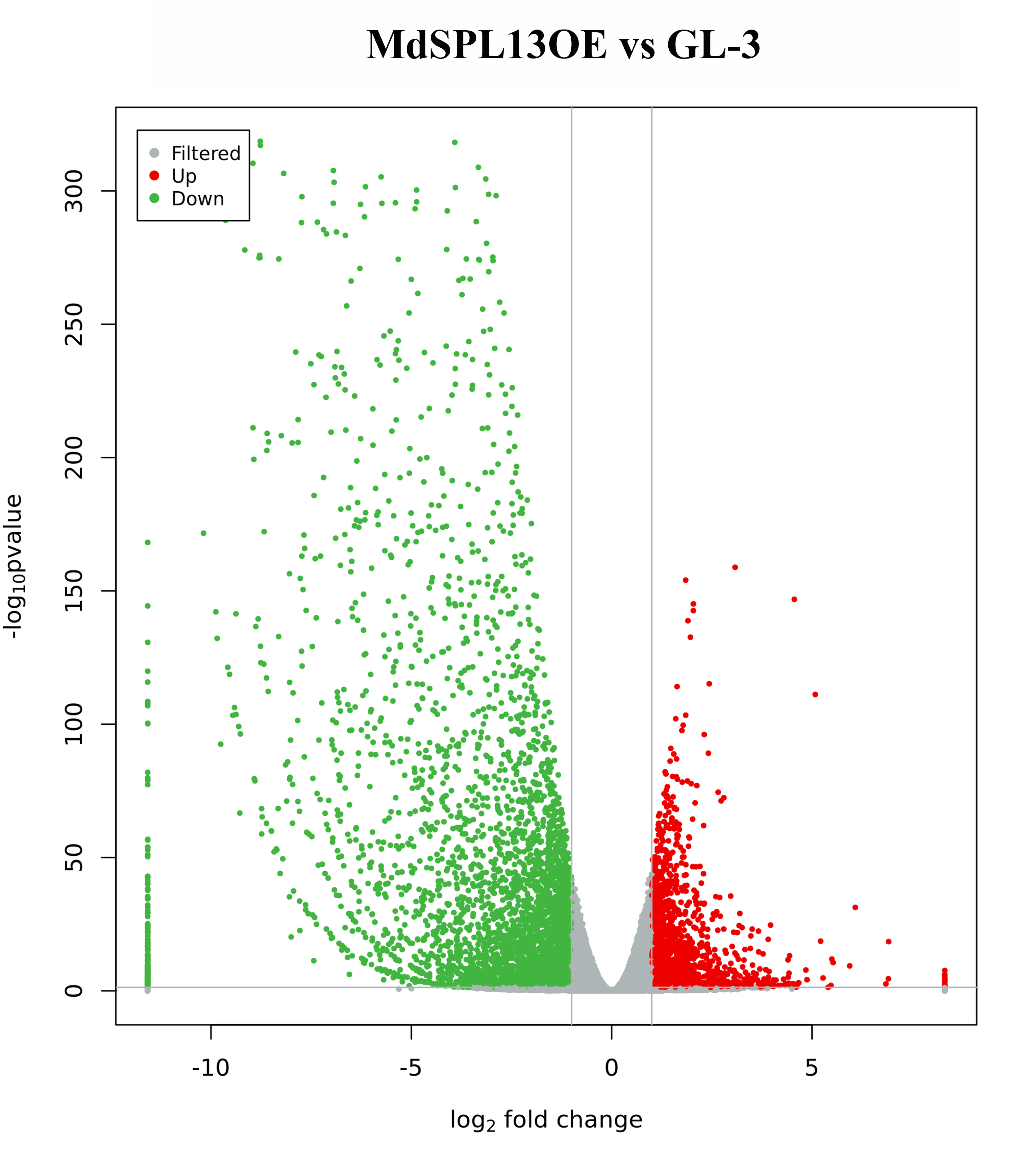


**Fig. S4** Volcano plot of DEGs identified by RNA-Seq.


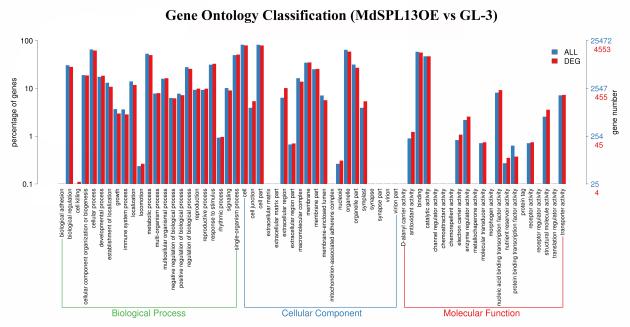


**Fig. S5** GO enrichment map of DEGs identified by RNA-Seq.


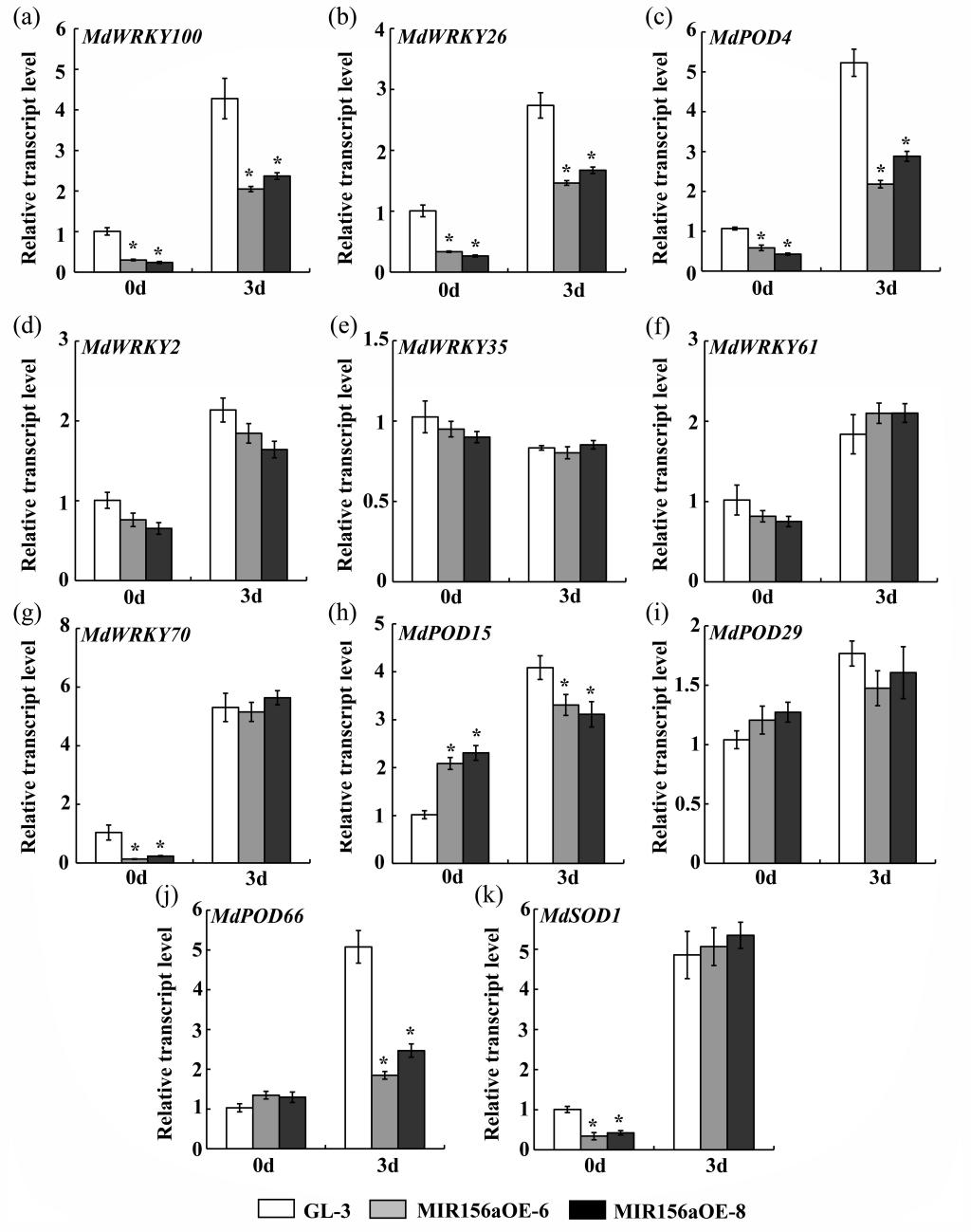


**Fig. S6** Differential gene expression analyses of ‘GL-3’ and MIR156aOE lines under salt stress treatment. Error bars indicate standard deviation (SD) for three biological replicates. Asterisks indicate significant differences between treatment means (*P* < 0.05; *t*-test).


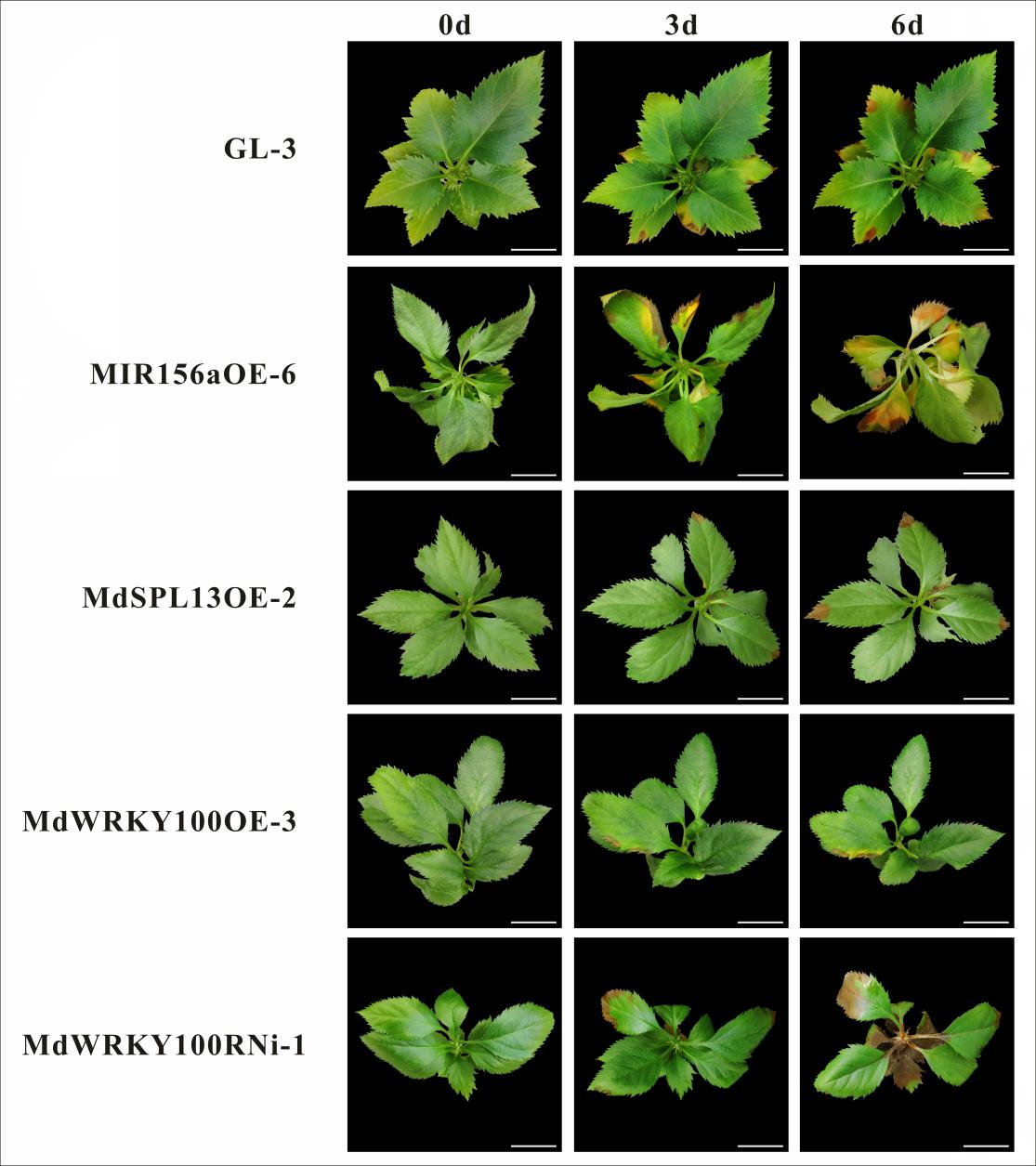


**Fig. S7** Phenotypes of ‘GL-3’, MIR156aOE, MdSPL13OE*,* MdWRKY100OE and MdWRKY100RNAi plants under salt treatment, scale bars = 1 cm.

Table S1 Primer sequences used for cloning *MIR156a* and *MdSPL13*

| Primer | Sequence（5ˊ→3ˊ） | Use |
| --- | --- | --- |
| MdSPL13-F  MdSPL13-R | CCCGGGATGGATTCAGGCAGAGCTCAT  CGGGATCCTTAATTAGACGTGCCT | Primers for *MdSPL13* full-length  sequence amplification |
| MdSPL13-S-F  MdSPL13-S-R | TCTAGAATGGATTCAGGCAGAGCTCAT  GGATCCGACGTGCCTCCATGTC | Subcellular localization primers for *MdSPL13* |
| MdSPL13-T-F  MdSPL13-T-R | GAATTCATGGATTCAGGCAGAGCTCAT GAATTCTTAGACGTGCCTCCATGTC | Transcriptional activity primers for *MdSPL13* |
| MIR156a-F  MIR156a–R | GTAGCGAGTGGTGGTGACAG GCGGCTGACAGAAAGAGAAG | Primers for *MIR156a* precursor sequence amplification |

Table S2 Primer sequences of RLM-5’RACE

| Name | sequence（5’-3’） |
| --- | --- |
| GeneRacer RNA Oligo | CGACUGGAGCACGAGGACACUGACAUGGACUGAAGGAGUAGAAA |
| GeneRacer^TM^ 5’Primer | CGACTGGAGCACGAGGACACTGA |
| GeneRacer ^TM^ 5’Nested Primer | GGACACTGACATGGACTGAAGGAGTA |
| MdSPL13-I Outer Primer  MdSPL13-I Inner Primer  MdSPL13-II Outer Primer  MdSPL13-II Inner Primer | CCTCAAACACATACAACTAAGAGA  GAAGAAAACAAGAATCATCATCAG  AAGCATGCTATGCATATTTGTTTG  GAATCATCATCAGCAACTGAACAG |

Table S3 Primer sequences of qRT-PCR analysis.

| Gene | Forward primer(5’-3’) | Reverse primer(5’-3’) |
| --- | --- | --- |
| RT-5s rRNA | GTCACATCGTATCGTGAAGCTGCGCAGCTGATGTGACTGGATTGG | |
| 5s rRNA MIR156a | TGCACTAGCGTGTAGAGGAACC TGCACTAGCGTGTGACAGAAGA | ACATCGTATCGTGAAG ACATCGTATCGTGAAG |
| RT- MIR156a | GTCACATCGTATCGTGAAGCTGCGCAGCTGATGTGACGTGCTCAC | |
| EF-1α | ATTCAAGTATGCCTGGGTGC | CAGTCAGCCTGTGATGTTCC |
| MdSPL13 | TCAGGCAGAGCTCATGGAAA | TCACCATCGTCACTCTTGCT |
| MdWRKY2 | GAGGTTTCAGTCCAGTCCGA | ACCGTATCGAAAGCCCTAGG |
| MdWRKY26 | CCAAGCCACAGAACCCTAGA | CTCCTCCTGACTTGCTCCTC |
| MdWRKY35 | CTAAAGAATGAAGGGCCGCC | TTGTTGGGTGGTGGAGATCA |
| MdWRKY61 | ATGCCAAAGAGGCCTTCAAA | TCGTTTTCTTCCGTAGCCTC |
| MdWRKY70 | GACCCCACATCACCAAAACC | TCCTCATCCCAGTGCATGTT |
| MdWRKY100 | CTCACACTCAGCAGCCTTCA | GAGGGAGGAGAAATGGGCAG |
| MdSOD1 | ACGTCCCTGAAGCTTTGAGA | ACTTCCTCCTTCACCAGCTC |
| MdPOD4 | TCTCACACAATTGGCCTTGC | TGTTCCGTTGAAGAGCTCCT |
| MdPOD15 | CAGTCCTGATCCGACCTTGA | CTTTGGAGCAAGCCTTCCTC |
| MdPOD29 | CCAAGTTCAGGGCTGTGATG | CCTGACACAACCACAGCTTC |
| MdPOD66 | GAGAAAGACGACCCTCCCAA | CTTCCGTCTTTCCTCCCCTT |
| MdSPL5 | CTCATCAGCAGTCGGCGTCA | TCGGGGTTAAGAGAAAGGGCAG |
| MdSPL8 | TTCACGGGTCATCCCTTGC | GCTGCTTTGCCTTTCTTGCT |
| MdSPL11 | TGGTGGACTTTGATAAGGTGAAGAGA | ACTTCGTTCGTCAGTTGCGGA |
| MdSPL16 | GGAGCATGAGAGAAACTTTTTTCAAGTA | CACATTGTTGCTTGCACGCC |
| MdSPL17 | GTCCTTGCCATGCCCGAT | TCAGGTTTTCCAGTTCCCACA |
| MdSPL24 | CCTCTGTTCAAAGCCGCAATCT | ACTGGACATAAAACTCCCCGGAT |
| MdSPL25 | GCCAAGAGATACTCCCTACTGACATTC | CTGGAACTGCTGAAGCTGTGTTG |
| MdSPL27 | CAAGATATTCGTGCGGATTCGATTAA | CTGTGGAAATGTTGGAATTCTGATGC |
| MdSPL28 | TCCCCTGATGTCCGAGTGCT | TTTCAGATGCGTGCCCCGAC |

Table S4 Primer sequences used for cloning promoters

| Primer | Sequence（5ˊ→3ˊ） | Use |
| --- | --- | --- |
| *MdWRKY26*-pro-F | TCGAGCTCGGTACCCGGGCTGGACTAAACATGATTATG | Clonging for *MdWRKY26*  promoter |
| *MdWRKY26*-pro-R | ATGCCTCGAGGTCGACTATAACATACGATAAAGTTT |
| *MdWRKY100*-pro-F | TCGAGCTCGGTACCCGGGGTATATTTTTAATGTTTCAC | Clonging for *MdWRKY100* promoter |
| *MdWRKY100*-pro-R | ATGCCTCGAGGTCGACAAATTAATAATATCATGTGA |
| *MdPOD4*-pro-F | TCGAGCTCGGTACCCGGGGGAGACTAGTTGGAGAAGGA | Clonging for *MdPOD4*  promoter |
| *MdPOD4*-pro-R | ATGCCTCGAGGTCGACATTAAATAATTTAATTAAAC |
| *MdWRKY100*-pro-P1-F | TCGAGCTCGGTACCCGGGTACATGCTTTTAGATAAATT | Clonging for *MdWRKY100* promoter P1 |
| *MdWRKY100*-pro-P1-R | ATGCCTCGAGGTCGACTTCAATAAGATTCATCCACC |
| *MdWRKY100*-pro-P2-F | TCGAGCTCGGTACCCGGGAGTGTGAATCTCACGTTGGA | Clonging for *MdWRKY100* promoter P2 |
| *MdWRKY100*-pro-P2-R | ATGCCTCGAGGTCGACAAATTATTAGGAGCCAATCT |
| *MdWRKY100*-pro-P3-F | TCGAGCTCGGTACCCGGGCTTTATAATATAAACATAAA | Clonging for *MdWRKY100* promoter P3 |
| *MdWRKY100*-pro-P3-R | ATGCCTCGAGGTCGACTTATATTGTAAGTCTGTTGT |
| *MdWRKY100*-pro-F1-F | TTATGTCTTCATTACTCAAGGTTTGGTACGGTAGATAACAACTTAAGCGG | Clonging for EMSA |
| *MdWRKY100*-pro-F3-R | CCGCTTAAGTTGTTATCTACCGTACCAAACCTTGAGTAATGAAGACATAA |
| *MdWRKY100*-pro-F1-probe | TTATGTCTTCATTACTCAAGGTTTGGTACGGTAGATAACAACTTAAGCGG |
| *MdWRKY100*-pro-F3-F | ACATAAAAATAAAAAAACAAATTGTACTAAAAATTATTACAAACTTTATC | Clonging for EMSA |
| *MdWRKY100*-pro-F3-R | GATAAAGTTTGTAATAATTTTTAGTACAATTTGTTTTTTTATTTTTATGT | Clonging for EMSA |
| *MdWRKY100*-pro-F3-probe | ACATAAAAATAAAAAAACAAATTGTACTAAAAATTATTACAAACTTTATC |
| *MdWRKY100*-pro-P1-F | AAGCTTTACATGCTTTTAGATAAATT | Clonging for LUC |
| *MdWRKY100*-pro-P1-R | GGTACCTTCAATAAGATTCATCCACC |
| *MdWRKY100*-pro-P2-F | AAGCTTAGTGTGAATCTCACGTTGGA | Clonging for LUC |
| *MdWRKY100*-pro-P2-R | GGTACCAAATTATTAGGAGCCAATCT |
| *MdWRKY100*-pro-P3-F | AAGCTTCTTTATAATATAAACATAAA | Clonging for LUC |
| *MdWRKY100*-pro-P3-R | GGTACCTTATATTGTAAGTCTGTTGT |
| *MdWRKY100*-pro-S1-F | TCGGTCATGTTACTTTCAAGTTCT | Clonging for CHIP |
| *MdWRKY100*-pro-S1-R | ACCGGCCATGATTGCTTTTG |
| *MdWRKY100*-pro-S2-F | AGGAAGCAAGAAAGGCACCT | Clonging for CHIP |
| *MdWRKY100*-pro-S2-R | TCTACCGTACCAAACCTTGAGT |  |
| *MdWRKY100*-pro-S3-F | AGGGAGTCAAAACAACCAATCA | Clonging for CHIP |
| *MdWRKY100*-pro-S3-R | TCATTCAACGAGTAAAGTCCAATACT |
| *MdWRKY100*-pro-S4-F | CCAACCAGTCAAACCCACGA | Clonging for CHIP |
| *MdWRKY100*-pro-S4-R | GGTCACGTATTGCACAAGGC |
